# In Plain Sight: Open Doors, Mixed-sex Wards and Sexual Abuse in English Psychiatric Hospitals, 1950s—Early 1990s

**DOI:** 10.1093/shm/hky091

**Published:** 2018-11-08

**Authors:** Louise Hide

**Affiliations:** Department of History, Classics and Archaeology, Birkbeck, University of London, Malet Street, London, UK

**Keywords:** mixed-sex ward, psychiatric hospital, sexual abuse, open doors, deinstitutionalisation

## Abstract

This article investigates the consequences of unlocking psychiatric wards and allowing male and female patients and staff to mix freely in the post-war period. I argue that the sexes were allowed to socialise with each other primarily for the benefit of male patients, and that some superintendents were ‘blind’ to the dangers of sexual abuse to which female patients were exposed, especially given the growing number of male ‘sexual psychopaths’ who were being admitted to open wards. While male nurses did complain about mixed wards in the mid 1960s, it was not until the rise of feminism and patient activism that the extent of sexual abuse and violence in hospitals began to be revealed a decade later. By the 1980s, despite calls to return to segregated living, psychiatric hospitals were no longer able to fund single-sex wards, exposing many women to sexual danger and deterring them from seeking help as in-patients.

## Introduction

In 1992, the mental health charity MIND published a policy paper titled *Stress on Women*, which was part of a nationwide campaign to end sexual harassment and abuse in mental health settings.^1^ Mixed-sex wards came in for particular criticism. Several initiatives had been militating against them in both general and psychiatric hospitals since the late 1970s, only 20 years after the practice had begun.^2^ One woman recounted:

I and a number of other women I know have had very bad experiences on mixed wards. A friend of mine was raped and I was harassed to death by men and the staff never intervened. The average male response was ‘It’s all your fault’. You only had to say ‘good morning’ and you were told this was a come on. The staff think women being there will make the men behave better and that if men behave badly it’s the fault of women. … Because of this appalling state of affairs, I couldn’t stay in hospital. Now when I feel very depressed, I stay with a friend. The situation in the hospital was so awful, I’d never go back there—you can’t be ill in peace.^3^

This woman’s testimony is important. Not only does it describe why some women with serious mental health problems avoided in-patient psychiatric care and treatment, but it evokes entrenched staff attitudes around women, sexuality and mental health which can be traced back to the late nineteenth century.

In this paper, I address the move towards mixed-sex wards and its implications in the post-war period from the 1950s to the early 1990s when significant changes took place inside Britain’s psychiatric hospitals. A growing body of historical research examines broader shifts from deinstitutionalisation, the anti-psychiatry movement and the therapeutic communities that were run by some maverick psychiatrists.^4^ Yet we know less about how services were developing inside the ordinary mental hospital. As Vicky Long has argued, post-war changes did not ‘rapidly eradicate institutional care’, but deinstitutionalisation did recalibrate practices and cultures inside hospitals.^5^ The rate at which this happened varied considerably between one establishment and another. For John Hopton, the social and cultural life of Prestwich Hospital remained virtually stagnant until it was stirred into action following the spate of abuse inquiries that were held in the early 1970s.^6^ Other more progressive hospitals such as Fulbourn, Claybury and Severalls, all of which were run by ambitious medical superintendents, introduced sweeping changes that were intended to rehabilitate institutionalised patients by creating a more ‘normal’ environment that would prepare them for life outside the hospital.^7^

Given the enormity of the reforms that took place in post-war psychiatric hospitals, many of which involved ill-considered social and therapeutic experiments, it is unsurprising that adverse consequences would have occurred. Entrenched cruel and dehumanising practices, such as locked wards and excessive medication, may have been eliminated, but new ones often replaced them. Patients were still harmed, but in more insidious ways and, to use a well-worn term, ‘in plain sight’. Abuse was seen, but not seen, reflecting broader social attitudes towards women and what constituted sexual harassment and harm.

In the vast historical literature on asylums and psychiatric hospitals, accounts of abuse and violence in their many forms lurk in the interstices of institutional systems and practices without being explicitly addressed in detail. We know that most staff and doctors were well-meaning and did not intend to hurt their patients. But patients were harmed, sometimes unintentionally, sometimes deliberately. While child abuse is the subject of a growing body of historical scholarship, less attention has been paid to adult care in long-term psychiatric institutions.^8^ Claire Hilton has begun to address a lacuna in the historiography with her work on ‘whistleblowing’ and how the reformer Barbara Robb contributed to the exposure of cultural and institutional failings that led to abuse, particularly on psychogeriatric wards.^9^ In *Destigmatising Mental Illness?* (2014), Vicky Long devotes a chapter to the portrayal earlier in the century of the male mental patient as ‘violent and brutish’, building on this argument in a subsequent essay where she explores how some male nurses attempted to prevent female nurses from working on male wards by emphasising the distasteful nature of the work.^10^ Male nurses made similar claims during the 1960s, concerned that their wards were being taken over by a ‘petticoat government’.^11^

Here, I set out to examine two important and interlinked post-war ‘innovations’ and their consequences: the ‘open-door’ policies in which many wards were unlocked allowing patients to move freely around the hospital and, sometimes, its environs; and the creation of mixed-sex wards. I argue that the rush to introduce these reforms during the 1950s was instigated primarily to benefit male patients and that the implications for female patients—their exposure to sexual abuse from male patients and staff—were ill-considered due to widely held beliefs around the relationship between women, sexuality and mental disorder. Furthermore, I contend that by the time the nature and extent of abuse began to be revealed in the mid to late 1960s and 1970s, the provision of psychiatric services had adjusted to a more cost-effective mixed-sex model, making a widespread return to segregation unaffordable.

This paper traces a broad chronological unfolding of events, which could explore a number of variables. I focus on the sexual abuse of women by male patients and staff in psychiatric hospitals, analysing such practices through attitudes towards female sexuality, mental illness and age. Space does not permit me to address the abuse of male patients, who were also subjected to sexual violence.^12^ Neither am I able to explore in detail the situation in general hospital wards or in institutions for people with intellectual disabilities, the latter of which draws on a different set of discourses and research.

Lastly, a few words on terminology. The term ‘sexual abuse’ as we understand it today did not begin to gain currency until the late 1970s. Following Lucy Delap’s work on child abuse, I use the term ‘abuse’ in order ‘to group together a range of historical sources that are only retrospectively read as concerning abuse’, but which may have been recognised as abuse even though they were framed in different terms.^13^ Here, the term ‘sexual abuse’ describes the sexual mis-use or harm perpetrated by one individual or group against another through, for example, forced or coercive rape or other direct acts of a sexual nature, or harassment through touching, exposing, staring, looking or making sexual remarks. In the context of people resident in psychiatric institutions, abuse and harassment are not only complex due to issues around mental capacity and consent, but incorporate other forms of abuse, including that of trust and power invested in staff.

## 1950s–1960s

### From segregation to integration

The segregation of the sexes had been encoded into asylum systems and structures since the early nineteenth century.^14^ Virtually every aspect of institutional life reinforced notions of sex difference—from the architecture to working practices, uniforms and clothing, dietary allowances and the daily routine in which blocks of time and space closely circumscribed patient movement, usually to keep men and women apart. Not only did this system apply to patients, but to nursing staff too. Female wards were strictly off limits to men, except for doctors and chaplains. Asylums had a strong moral purpose and there were few more powerful indicators of institutional failure than when a female patient became pregnant whilst in detention.^15^

Some relaxation of the rules around segregation, particularly in regard to posting female nurses onto male wards, began to take place during the late nineteenth century.^16^ Yet wider change for patients did not come until the passing of the 1930 Mental Treatment Act, which allowed voluntary admission to and, therefore, discharge from hospital. As a result, some institutions began to adopt a less custodial character and permit selected patients to move around the hospital beyond their wards. When war broke out in 1939, hospitals became severely short staffed and patients were once again locked inside desperately overcrowded and under-resourced wards.^17^

After the war, many countries in Europe and the USA turned their attention to the home front where they were confronted by the shocking state of public mental hospitals. Having been de-mobbed from the army, Eric Pryor joined Claybury Hospital as a student nurse in 1948. He likened the wards to a ‘Dickension [*sic*] workhouse’, describing a regimented discipline of staff clocking in and out, drab ‘state-issue’ patient clothing, and a huge building in a state of ‘war-wounded shabbiness’.^18^ Overcrowding was a serious problem, much of which was due to an accumulation of older long-stay patients. By the end of 1954, more than 152,000 patients were under treatment and living in vast and often dilapidated buildings in England and Wales.^19^ Around 46 per cent of patients had been resident for over 10 years, of which 24 per cent had been resident for over 20 years, and of this number 10 per cent for over 30 years.^20^

Following the revelation of the shocking atrocities that had been perpetrated during the Second World War, particularly in concentration and prisoner-of-war camps, a new impetus to turn hospitals into more humane environments quickly gathered pace.^21^ It was reinforced by a 1953 report published by the World Health Organization, which stated that:

The life within the hospital should, as far as possible, be modelled on life within the community in which it is set. In a western country where men and women mix freely at work and in recreation, it is obviously desirable that they should do so when in the mental hospital.^22^

By this time, social scientists in Europe and the USA had already begun to turn their attention to the institutional environment and study its effects on the lives of patients and staff. The American sociologist Morris S. Schwartz and psychiatrist Alfred H. Stanton argued in *The Mental Hospital* (1954) that the hospital should be understood as a social system and the hospital ward as a social world.^23^ In Britain, Russell Barton, the medical superintendent of Severalls Hospital, published a book entitled *Institutional Neurosis* in 1959. The culture of the mental hospital was, he argued, pathogenic in its own right. Many patients who had spent long periods of time inside mental institutions suffered from two disorders: the original illness—often diagnosed as schizophrenia—and another condition caused by living in the institution.^24^ Framing the effects of the institution as a condition or a pathology and giving it a name—‘institutional neurosis’—transformed it into a potentially treatable condition, offering psychiatrists something tangible to work with: if the underlying cause of institutional neurosis was environmental, patients could *learn* through therapeutic means how to live like ‘normal’ adults and take responsibility for their own actions. Ultimately, they could leave hospital and reside in the community.

Desperate to move patients out of the costly and out-dated asylum system into community living, both Conservative and Labour governments were quick to endorse a more therapeutic environment that would facilitate the rehabilitation of long-stay institutionalised patients. The shift in responsibility for mental treatment from local government to the newly-formed National Health Service in 1948 reinforced political resolve by extending the reach of out-patient care to clinics, GP surgeries and psychiatric units.^25^ But it took decades to dismantle the great asylum behemoth and to shift care from the institution into the community, mainly due to the failure of successive governments to provide sufficient funding and services to facilitate the transition. Indeed, the old asylums did not begin to close their doors until the 1980s.^26^

Meanwhile, changes needed to be made inside hospitals in order to prepare male and female patients for life outside by habituating them to socialising with each other and to live more freely in an open environment. The first move involved unlocking ward doors, as well as removing bars from windows and taking down fences in the old prison-like airing courts. Dingleton Hospital in Scotland was the first to be fully open in 1949, followed by Mapperley Hospital in Nottingham in 1953.^27^ Other institutions followed their example, encouraged by the arrival of new phenothiazine drugs that could control psychotic symptoms as well as violent and aggressive behaviour. The passing of the 1959 Mental Health Act which, crucially, removed the necessity for a magistrate to approve the compulsory detainment of a patient added to the move towards decarceration. From this moment on the power of confinement passed primarily into medical hands. Patients who voluntarily went into a psychiatric hospital did so on the same informal basis as they would enter a general hospital and were at liberty to discharge themselves at any time.^28^

The basic principle behind ‘open-door’ policies could not have been more straightforward, yet it represented one of the most radical institutional changes to take place in the post-war period. Huge risks were involved in what were, in effect, social experiments. Most open-door policies were instigated by individual medical superintendents who had to persuade uneasy staff and people living in the surrounding communities that allowing patients to move freely around the hospital and into its local vicinities was a good idea.^29^ However, anxieties that a dramatic removal of institutional fetters would set patients on a course of violence and destruction never materialised. Indeed, opening wards appeared to reduce violence. At Warlingham Park, which was unlocked from 1954, Dr Rees claimed that open doors relieved the nurses of having to play the jailer, leaving patients less resentful and angry.^30^

In the 1950s, most wards remained single-sex but unlocked. Male and female patients were at liberty to leave their wards and to meet each other unchaperoned in communal areas of the hospital. They would mix during mealtimes, and when watching television and taking walks together.^31^ At Warlingham Park, men and women, both voluntary and certified, mixed freely in the canteen and grounds, with one commentator noting in 1954 that ‘no harm (though much good) has ever come of it’. Many patients claimed that a turning point in their illness had taken place when ‘some patient of the other sex took a friendly or even sentimental interest in them’.^32^ J. C. Barker, a psychiatrist at Shelton Hospital, wrote that when men and women were brought together for meals and recreational activities, the men ‘smartened themselves up in the presence of females, and many patients who for years had hardly spoken to members of their own sex began to take an active interest in each other and in their hospital’. Integration did not, he insisted, result in any increased promiscuity.^33^ A charge nurse from Moorhaven Hospital in Devon, R. J. Roberton, described how mixing the sexes resulted in a reduction in spitting and incontinence, as well as in acts of violence and aggression. ‘The decrease in noise was noticeable, likewise the eternal pacing and general restlessness of the men’, he noted, before adding: ‘The presence of women moving among them and helping them in their ward routines acted as an inspiration and a spur to improving their behaviour. … One hopes that this mixing will reduce the tendency to homosexual behaviour and divert relationships into more normal channels’. Roberton extolled the new ‘therapeutic’ practice as positive and ‘revolutionary’.^34^

In his work on therapeutic communities, sociologist Liam Clarke has provided some historical background to the practices and consequences of open-door policies, including the antagonism that they caused between male nurses and doctors, described below. Yet he has given less consideration to what, with hindsight, was an inevitable consequence of allowing male and female patients and staff to mix, which is the increased potential for sexual assaults to take place in unsupervised and empty hospital spaces.^35^ Indeed, I have found very few debates on this in the medical literature from the 1950s where such risks were either not adequately considered or only vaguely alluded to by medical superintendents.

### Mixed-sex wards and the ‘psychopathic offender’

The potential for and prevalence of sexual abuse in psychiatric spaces did not, I argue, become openly apparent, or at least impossible to ignore, until the 1960s when the composition of the in-patient population began to change. As more people were treated outside psychiatric institutions and many long-stay patients began to be re-settled in the community or in private residential homes, the hospital population fell steadily after 1954, even though by 1970 admission numbers had grown by 17 per cent.^36^ From the early 1960s, hospitals began to develop two different types of patient constituency, which not only complicated systems of care but brought together people who subscribed to different social values. Around 30 per cent of residents were over 65, many of whom had spent years in the institution; the second group was formed of men and women who were admitted, often repeatedly through the ‘revolving door’, for a short period of time during the acute stage of their illness or to have their medication monitored.^37^

At around the same time, mixed-sex psychiatric wards—where men and women lived on the same ward but did not share a dormitory—began to open in order to bring the sexes together in a ‘normalised’ social environment. This was especially the case for those who were preparing to move into the community following decades of institutional living. Peter Cheng, a charge nurse on one of the first mixed-sex wards at Severalls Hospital in Essex, believed that integrated living was crucial to the process of discharging people from hospital into supervised community homes. He explained that ‘it would be too much for them to actually go straight out … not only having to get used to the … different kind of rigid routine, but having different kind of people, different gender of people’ [*sic*].^38^

Selecting the right patients for admission into unlocked psychiatric wards as well as for discharge into the community was crucial to the success of new open-door policies. But as the material boundaries inside hospitals disappeared, there was growing anxiety around ‘criminal psychopaths’ who were being admitted to open psychiatric hospitals due to the lack of suitable secure psychiatric facilities in special hospitals such as Broadmoor, Rampton and Moss Side. An increasing number of men, some of them sex offenders, were admitted from courts and prisons into open psychiatric hospitals where they mixed with both male and female patients but had little interest in treatment.^39^ As a group these ‘offender-patients’ were unpopular in the hospitals, disliked by staff and patients alike. Their presence on wards was disruptive and they were frequently held responsible for sabotaging the painstaking therapeutic ward work carried out by doctors and nurses.^40^ David Clark, the medical superintendent of Fulbourn Hospital, described in 1956 the problems of dealing with the prisoner-patient, who usually ‘hangs about for months in the admission ward, watched nervously by the staff lest he commit some antisocial act’.^41^

One of the great challenges facing psychiatrists like Clark was controlling patients’ movements without resorting to restraints, seclusion and drugs. It was not unusual for patients to be kept in pyjamas, their clothes confiscated to prevent them from leaving the ward. Clark warned that ‘there is a certain danger that too much emphasis on open doors may lead to patients being too heavily sedated, subjected to too much physical treatment, or kept too long in bed’.^42^ Many hospitals kept one or two locked wards for particularly ‘troublesome’ patients, who were disproportionately women. In 1957, Harold Maddox wrote in the *Nursing Mirror* that in one hospital 34 per cent of female patients ‘were in “disturbed” or “refractory” wards with only 17 per cent of men in a similar position …’.^43^ At Claybury, the physician superintendent Denis Martin kept one locked ward for women who were ‘promiscuous by reason of mental illness *alone*’, whilst on the male side he reported that ‘such patients are not nursed in a locked ward but spend their day in small groups closely supervised by a nurse’.^44^ A Severalls psychiatrist explained that ‘the female side was always far more closely regulated because there was always the fear of pregnancies, always the fear that someone might have tampered with a woman’.^45^

What, then, of women who were not kept in locked wards but were still vulnerable to sexual abuse and violence, particularly if they were heavily medicated or experiencing the after-effects of treatments such as ECT? At Severalls, patients would meet in the underground passages that accommodated the ducts: ‘there [were] other sort [*sic*] of sex scenes going on’, the psychiatrist there recalled in the mid 1990s. ‘Some of the women had grounds privileges—not many of them—but after they’d had electrical treatment they could quite easily be screwed, and there were plenty of bushes and places to take them’.^46^ One woman who had been a patient there during the 1970s recalled her own horrific experience:

It was then I got raped. Somebody’d been to prison and was put in a psychiatric ward and he systematically made love to somebody who was married—made love to me—he was generally very sexually over the top. … He took me out into the grounds into some ward that was being redone up. It *was* rape. Because I … would never have done it. But I was high … ECT makes you peculiar … I was on ECT then. It was a very spaced-out time.^47^

The new open-door policies and the growing presence of sexual psychopaths in open hospitals particularly exercised male nurses, inflaming festering resentments that were rooted in the poor esteem in which they were held. Men were neither admitted to the general register of nurses until 1949, nor permitted to join the Royal College of Nursing until 1960.^48^ Psychiatric nurses were frequently under-represented in high level consultations, such as those conducted for the ‘Salmon Report’.^49^

While the views and opinions of psychiatric nurses were rarely sought at a higher political or organisational level, they held a great deal of sway on the wards. Male nurses were often well respected because they tended to be older, remained in post for longer, and were believed to add a general sense of stability to hospital environments.^50^ Many, especially those with long service records, opposed the practice of allowing the sexes to mix, seeing it as a major threat to both the status quo of ward cultures and to the somewhat autocratic methods used to run them. They resented doctors reorganising the social life of the hospital, which they, the nurses, would be required to manage. Some male nurses accused doctors of pursuing vanity projects at the expense of patient safety. Denis Martin probably did not help matters when his book on therapeutic communities at Claybury was published in 1962 under the title *Adventure in Psychiatry*.

Matters came to a head at the 1965 Confederation of Health Service Employees (COHSE) conference in Aberdeen when the tenor and content of the speeches reached such a pitch that the more salacious elements were reported by the mainstream press. Male nurses took to the platform, accusing psychiatrists of implementing mixed-sex wards in a ‘haphazard’ manner and of experimenting with patients to present themselves as progressive. The blame, *The Sun* newspaper reported, was laid ‘squarely on the shoulders of psychiatrists seeking the limelight by trying to be first in a fashionable field’.^51^ The General Secretary proclaimed:

We believe that before any consultation is undertaken by any psychiatrist in the country, and I do not care how many letters he has after his name or what is his standing, it should be realised that the mental nurse knows a sight more about the patient than does the psychiatrist. (*Applause*).^52^

Jack Charles, a member of COHSE’s National Executive Committee, made an impassioned speech: ‘most of you have wives and daughters and none of them is immune from having a mental breakdown’, he remonstrated. ‘They may have to enter a psychiatric hospital for treatment and while there they will be exposed to the risk of being seduced by that type of person’, whom he described as ‘deadbeat, layabout psychopaths’.^53^ Charles went on to report incidents of ‘blatant cases of sexual intercourse … and of the pregnancies which have been terminated’, adding ‘I know of a situation where a known prostitute was practising her profession inside the hospital’.^54^ There were reports of ‘sex orgies’ on wards, and of women being put on the contraceptive pill at Storthes Hall in Yorkshire.^55^ Questions were also raised around allowing violent patients who had been sent from prisons and courts to be placed on open wards with ‘young decent schizophrenics’.^56^ One delegate commented that patients—especially women—were exposed to blackmail by ‘criminal and general layout types’ having done or said degrading things on the ward during an acute stage of their illness.^57^

In response, William Ross, Secretary of State for Scotland cautioned COHSE ‘not to fall into the danger of over-exaggerating their allegations of sexual orgies in mental hospitals’.^58^ Dr Thomas Reading of Cefn Coed Hospital in Swansea claimed that the ‘prostitute in the corridors allegation was a “harmful distortion”’. The medical superintendent of Craig Dunain Hospital in Scotland insisted that ‘the behaviour of the patients in general is good’.^59^ Psychiatrist J. C. Barker commented that he could see how ‘the rapid introduction of integration may tend to undermine the authority of some nurses, particularly those who are personally insecure’.^60^

The media reports stoked a good deal of public anxiety around the perils of mixed-sex wards, particularly as relationships struck up between patients in hospital could potentially threaten marriages. Yet even though delegates at the Conference called for the Minister of Health to stop the practice ‘pending a thorough investigation at national level’, little action appears to have been taken.^61^ In 1969, COHSE was still agitating against the system, demanding that dangerous patients should be admitted to well-staffed single-sex psychiatric wards until regional Special Hospitals had been built.^62^ This latter move did not begin in earnest until after the Butler Report was published in 1975 calling for the creation of regional forensic facilities. By the late 1970s, a charge nurse was still protesting that not enough thought was given to who should and should not be allowed onto a mixed ward, claiming ‘You could find chronic alcoholics, drug addicts, sexual offenders or men with violent tendencies there’.^63^

### Male staff, female wards

In the 1950s and 1960s, more active treatment, open doors, a faster turnover of patients, and an increasingly ageing in-patient population intensified the demand for nurses.^64^ While some male nurses disliked the idea of working with the opposite sex, others took advantage of promotional opportunities on women’s and mixed-sex wards where senior posts were offered to prevent the attrition of valued male nurses.^65^ We know little of how female nurses responded to these changes, or of how female patients reacted to a more consistent male presence on the ward. It is possible that some nurses welcomed the move, believing that working alongside men would augment their own status.

Many male nurses were wary of working in close proximity with female patients and worried about, in the words of Denis Martin, ‘charges of sexual interference being brought against them by hysterical women patients’.^66^ Divisional nursing officer David Sharpe wrote in 1979 that ‘It is not unusual for a female patient, often young and attractive, to allege a male nurse has assaulted her’.^67^ Time and again, male nurses were exhorted not to remain alone on women’s and mixed wards without a female chaperone.^68^ If a male member of staff was discovered to have had sexual relations with a female patient, he could face a prison sentence.^69^

While false allegations of sexual impropriety were occasionally levelled against male staff by female patients, it is worth noting how little was made of the potential threat that male staff might pose to female patients. This was a delicate matter that tended to be alluded to in written accounts rather than being explicitly spelled out, possibly so as not to deter men from pursuing a nursing career or to reinforce negative stereotypes of male nurses. Even in 1977, one London health authority commented obliquely that the disadvantages of mixed-sex psychiatric wards were the ‘public misconception—promiscuity’ and ‘staff attitudes—vital to ensure that the “right” type of staff are appointed to work on mixed wards’.^70^

The unpalatable reality was that women on mixed-sex wards *were* sexually abused by male, and sometimes female, staff and patients.^71^ A social worker at Fulbourn Hospital in 1967 recalled that ‘there were occasions on which friendships … between staff and patients, which were highly inappropriate, could develop. And I think patients were sometimes very confused—even more, families were very confused’, she said.^72^ In principle, superintendents did condemn sexual relations between patients and staff.^73^ However, when asked who was ‘going’ for the women patients, the Severalls psychiatrist answered: ‘Some male staff. Sometimes it was the nursing staff, but quite often it was the artisans, who thought these girls needed a good time. I must tell you’, he continued, ‘I had some sort of sympathy with them, except that they weren’t necessarily consenting, and that—that’s a bit dirty’.^74^

### Gender, mental disorder and sexuality

Before exploring the major shifts that took place in the 1970s, I will consider why hospital superintendents appear to have been oblivious to the dangers female patients faced when doors were unlocked in the 1950s and 1960s. The most obvious reason is that post-war psychiatrists were desperate to liberalise hospital spaces and if they drew too much attention to the likely consequences of allowing the sexes to mix their endeavours to introduce therapeutic methods would have stalled, slowing the progress of deinstitutionalisation. There was also the matter of professional hubris and ‘seeking the limelight’ as argued so furiously by male nurses who, given the degree of influence that medical superintendents wielded in hospitals during this period, had a point. One psychiatrist interviewed by John Turner and his colleagues commented that until the 1970s, ‘services were dominated institutionally and intellectually by psychiatrists. We thought we were the experts and we decided’.^75^

There were more subtle reasons at play, too. As stated above, there was some acknowledgement that, in the words of Denis Martin, ‘a small … amount of promiscuity occurred’ when men and women socialised with each other. He then all but exonerated himself from taking any responsibility for it by adding that ‘it is very difficult to know what relationships are developing. We can only take steps when an undesirable association comes to our notice’.^76^ He did, therefore, accept that some male and female patients had sexual relations with each other, and an ‘undesirable association’ could have meant an abusive relationship. But by keeping his terminology vague, Martin was responding directly to public concerns around the threat of mixed-sex wards to marriages, rather than overtly drawing attention to the possibility that sexual violence or abuse might have occurred.

One explanation for a general ‘blindness’ to such events might be doctors’ ignorance of, or aversion to, non-normative sexuality that occurred outside marriage. In 1969, a Dr Malcolm Potts stated that ‘Doctors are confused about sex. … Most people get their family planning advice not from their doctor but from their barbers’.^77^ In her survey of English hospitals conducted during the early 1960s, journalist Gerda Cohen suggested that doctors found discussions around sex distasteful and embarrassing. At a weekly case conference that she attended, a nurse observed that the male patient in question—‘the case’—‘[has] got a crush on that psychopath in Twelve. … She won’t do him any good, a right tart’. When the junior housemen responded with a ‘stifled titter’, the nurse replied, ‘She’s a nymph isn’t she?’, bearing out another fear around promiscuous women assailing male patients.^78^ Nurses were often ignorant of sex, too. Even by the 1980s, they were given little training on the subject and found it difficult to discuss in terms that were anything but negative.^79^

A second reason why superintendents might not have considered the dangers of open doors is predicated upon them not ‘seeing’ some women patients as sexual and, by association, potential victims of abuse. A high percentage of both male and female patients had lived inside the institution for years, if not decades, and been rendered all but invisible by the experience. Russell Barton explained how institutional neurosis is ‘a disease characterized by apathy, lack of initiative, loss of interest … submissiveness … lack of individuality and sometimes a characteristic posture and gait’.^80^ On opening his book the reader is met with a panel of black and white photographs of women of indeterminate age, standing hunched, arms folded, wearing standard issue floral dresses and aprons, not looking directly into the camera but down, or away—evidence, in Barton’s view, of how the person had become the mental patient, an individual without an identity, or a sexuality, and therefore, unseen.^81^ This view was expressed in 1976 when the Chairman of the Kingston Richmond & Esher Community Health Council, Mrs M. E. van Embden, insisted that at one hospital:

wards can no longer even be termed ‘mixed sex’ but ‘asex’. That is to say the geriatric patient is seen not as an old man or an old woman but as a separate and sexless species. … It is presumed to be ‘alright’ to allow men and women over the age of 65 to share sleeping and toilet accommodation on the basis that they are no longer sexually aware.^82^

Third, in order to understand attitudes and subsequent behaviours of male staff, we must examine contemporaneous views around the relationship between mental disorder, women and sexuality. Some women whose so-called pathology had been linked to their sexual behaviour were labelled ‘feeble-minded’ earlier in the twentieth century. By the 1950s, they were being re-categorised as ‘severely subnormal’ or, if they had worked as prostitutes, they might be given a ‘psychopath’ diagnosis.^83^ This latter category had emerged from a set of discourses that gained traction in the late nineteenth century when connections were made between ‘sexual need’ and mental disorders such as neurosis, hysteria, erotomania and nymphomania in women.^84^ A woman’s unstable emotional state was believed to be linked to the vagaries of her reproductive system and the critical events taking place within it such as menstruation, the post-partum period and menopause. It was during this time that proponents of the new ‘scientific’ discipline of sexology were shifting the focus away from ‘aberrant’ sexual acts towards the individual, constructing and pathologising new sexual identities around ‘deviant’ behaviour such as the masturbating child, the hysterical woman and the homosexual.^85^ Following Freud, nymphomania began to be constructed as a symptom of a psychological rather than a biological condition, allowing it to be reframed as a neurosis that could be treated with psychotherapy.^86^

In the 1950s, so-called psychopathic women were seen as ‘higher grade’ patients in need of control rather than support. According to the 1957 Royal Commission report, they were among the most problematic of female patients, presenting a particular threat to male staff—men, that is, who had been taught to watch out for young girls and women, particularly ‘hysterical’ women, who maliciously ‘cried rape’ to achieve their own nefarious ends.^87^ Mentally disturbed women—the ‘hysterics’ and ‘neurotics’—were still believed to conjure up false stories of rape and abuse, sometimes as a result of an erotic delusion or during moments of excitement, such as when menstruating.^88^ Joanna Bourke has argued that these fears grew in the 1960s with the emergence of the ‘sex kitten’, described by one judge as young girls who allegedly knew more about sex than prostitutes.^89^ A little later in 1988, an anonymous psychiatrist described how an ‘acutely psychotic woman … claimed to have been raped in a [hospital] lift’. The patient was not fully believed until her ‘story’ was corroborated by several other patients, including ‘a reliable, married, intelligent, currently well outpatient’ who also reported to have been sexually molested in the lift whilst acutely ill.^90^

The psychiatrist’s association of credibility with psychopathology, character and social status—‘reliable, married, intelligent’—highlights the categorisation of female patients whose sexual promiscuity was interpreted not as a consequence of a faulty heredity or of being innately immoral but, as mentioned by Denis Martin, ‘by reason of mental illness *alone*’.^91^ In his 1956 annual report, David Clark complained that until some ‘radically new approach’ could be found to the ‘problem of the aged in need of institutional care’ Fulbourn Hospital would be unable to do ‘the work for which it is especially equipped—the treatment of the psychiatric breakdowns of those capable of returning and contributing to the community—the young housewives and the middle-aged mothers of families’.^92^

Both Martin and Clark were referring to a group of women who both conformed to gender stereotypes and were considered ‘treatable’. This made them more visible, more worthy of psychiatrists’ attention, and subsequently more protected than the ‘chronically untreatable’ patient. Being visible often meant being sexually attractive and women—both staff and patients—were judged by their physical appearance. In his memoir of Fulbourn, Clark described how the patients who were selected by the doctors as suitable for psychotherapy ‘were nearly always attractive and well-spoken young women’.^93^

To end this section, I wish to explore in a little more depth some of the points touched on above around the intersecting discourses between gender, sexuality and mental illness. Bodies are discursively constructed as sexual.^94^ They can also be constructed as *asexual*, or they can be *de*-sexualised creating a culture in which society, including ignorant and professionally isolated doctors, might not see potential dangers faced by certain female patients. In 1965, the psychiatrist at Shelton Hospital insisted that the integration of the sexes did not result in any increased promiscuity, even though other doctors such as Denis Martin contradicted him.^95^ As I have shown, doctors and nurses tended to understand sexual harassment and abuse within the mores of the day, which focused on unwanted pregnancies, the break-up of marriages and the exposure of ‘respectable’ women to blackmail following promiscuous behaviour during a ‘manic’ phase. All of these events were framed within the social role of women and refracted, I argue, through a prism which enabled male doctors to pursue their own professional interests while attending to standards of social morality, keeping the press and public at bay. Before the mid 1970s, sex between patients was discussed in relatively benign language, not in terms of violence and harm. These attitudes have been described by sociologist Stanley Cohen as ‘not literal denial but cultural interpretations and neutralisations which encourage a dulled, passive acceptance of violence: this is what men are like, this is the fate of women …’.^96^

Doctors could not deny that female patients were sexual beings, but in their view they failed to exhibit ‘normal’ sexuality. The female sexual psychopaths and mentally deficient were seen as pathologically amoral, their unbridled sexuality was hardwired into their physiological, psychological and social make-up. Like the ‘prostitute’ who was reported by the male nurses to be practising her profession in the hospital corridors, many women moved freely around the institution, bearing out Joanna Bourke’s claim that ‘sexually active women become “common property”’.^97^ Psychiatrists had little to do with old and ‘senile’ women, who were rendered invisible partly by the institutional environment and partly because they were no longer considered to be in need of treatment. By contrast, hypersexuality might be interpreted as a temporary *symptom* of the more treatable illnesses experienced by wives, daughters and mothers who were protected in locked wards until the acute stage of their condition had passed and they could be discharged back to family life.

## 1970s–1980s

In this second section, I examine some of the shifts that took place between the 1950s–60s when open doors were heralded as a therapeutic breakthrough and the 1970s–80s when calls to revert to single-sex wards began to grow louder, stoked by campaigns led by newspapers and organisations such as the Royal College of Nursing and the National Federation of Women’s Institutes.^98^ Much of this opposition was directed at general hospitals, which had also introduced mixed-sex wards into intensive care units in the early 1960s because the cost of equipping a separate male and female unit was prohibitive, and, according to journalist Gay Search, because men and women were ‘too ill to be aware of who was in the bed opposite’.^99^ Unlike psychiatric wards, there was no powerful therapeutic ideology behind this move. The advantages were, primarily, greater clinical expediency as well as cost savings. Unsurprisingly, the practice spread and it was not long before psychiatric hospitals also grew accustomed to the financial benefits of mixed wards. In his defence of this system, a Professor Calnan stated in 1978 that a return to single-sex wards would lead to ‘as many as 12,500 empty beds in England and Wales every day’. He added, rather sneeringly, that ‘prudery could cost us over £4 million weekly’.^100^

In 1977 and 1979, in response to growing media pressure and public alarm around mixed-sex wards, two surveys were conducted by the Department of Health and Social Security (DHSS) on bed usage in mixed-sex wards in general and psychiatric hospitals. The responses, which related mainly to general hospital stays, were varied. Many patients liked being in mixed-sex wards because it felt more ‘normal’ and less claustrophobic than a single-sex ward. Mixed day areas were still seen as beneficial and improving of ‘social behaviour, personal hygiene and appearance’ for those in long-term care such as ‘mental handicap’ and psychiatric facilities. Most complaints related to privacy and dignity, especially when it came to members of the same sex being required to share washing and toilet facilities.^101^ Older women patients found it particularly disturbing. One male nurse working on a psychogeriatric ward commented that ‘many elderly female patients, even if confused, resent being in a state of undress if a man is around’.^102^ Relatives complained too, often on behalf of female patients who did not like sharing toilet and washing facilities with men, or want to see them wandering around in ‘gaping pyjamas’. Some husbands found the prospect of their wives mixing with men on psychiatric wards particularly objectionable.^103^ Added to family concerns were cultural sensitivities. The *Stress on Women* campaign reiterated time and again how women from Asian and orthodox Jewish communities would not accept admission to a mixed ward, the implication being that such an eventuality would deter them from seeking help.^104^

The political impetus to investigate systemic abuse and neglect was further galvanised by the fall-out from a number of press reports and public inquiries into abuse in psychiatric and mental handicap hospitals in the early 1970s.^105^ They mobilised an increasingly powerful body of patient activism, as well as a scramble to employ more doctors and nurses who were to be trained to higher standards.^106^ Added to this was a challenge to medical authority from the new counter-culture movements.^107^ Together with the rise of feminism which raised public awareness around sexuality, these movements exposed deeply entrenched systems of denial and silencing.

### Feminism

In 1970, an influential article by Inge K. Broverman and her team of psychologists revealed how medical professionals judged patients’ behaviours and attitudes according to gender stereotypes, thus framing them as deviant and pathological, or normal and acceptable. The authors concluded that women were faced with the stark possibility of either having to conform to stereotypical feminine behaviour, or having their ‘femininity questioned’ and being perceived as deviant if they freely expressed characteristics that were seen as ‘male’.^108^ This was one of the central arguments developed by feminist psychologist Phyllis Chesler whose book *Women and Madness* (1972) launched a searing indictment of American psychiatry as a patriarchal enterprise in which women were pathologised, hospitalised, over-medicated and ‘treated’ both for conforming and for not conforming to the traditional female role. ‘What we consider “madness”’, Chesler wrote, ‘whether it appears in women or in men, is either the acting out of the devalued female role or the total or partial rejection of one’s sex-role stereotype’.^109^ Chesler’s work was followed by a raft of books by feminist historians, sociologists and psychotherapists who argued that mental illness in women was a patriarchal construct, or a product of their socially prescribed roles.^110^

Feminists also began to provide a new framework in which the many forms of non-consensual sexual behaviour could be conceptualised and articulated. Incest started to be framed as child abuse from the 1970s, and its prevalence began to be uncovered.^111^ Children and certain women, including those with intellectual disabilities, began to be seen not as manipulative temptresses, but as victims. Added to this was an increased awareness of the degree to which rape and sexual assault caused severe psychological trauma and harm.^112^ No longer could clinicians and administrators ignore research into the high proportion of women diagnosed with a mental disorder who had been subjected to sexual abuse in childhood. A survey that was carried out in the USA during the 1970s estimated that between 17 and 28 per cent of women had been sexually abused by men before puberty.^113^ Another reported that almost half of male and female in-patients had been sexually or physically abused, or both.^114^

‘Speaking out’, whether to a trained professional or to a concerned lay person, demanded enormous courage and abused women still feared that they would not be believed, particularly if they had been diagnosed with a mental illness.^115^ The increase in the employment of nurses and consultant psychiatrists, including female doctors, during the 1970s did result in better standards of care. By 1990, just over 25 per cent of psychiatrists were women, which made it easier for other women who had been subjected to abuse to seek help, as well as to expose the sexual exploitation that had been going on between some male psychotherapists and their female clients.^116^

One of the important achievements of feminism was to provide women and men with conceptual frameworks and a vernacular vocabulary that could help them to give meaning to and articulate how acts of sexual violence had been perpetrated against them. Joan Busfield wrote that ‘women … have often had very narrow definitions of rape and have been reluctant to define sexual intercourse with friends or spouses under coercion as rape’.^117^ Lucy Delap has argued that there was a language in which abuse could be articulated before the late 1960s, but it was more ‘easily adopted by policy makers and commentators than by victims and survivors’.^118^ From the 1960s, reports of rape did begin to increase, partly as a result of better reporting facilities, partly due to the encouragement and support of feminists.^119^ Yet many psychiatric in-patients—especially those on long-stay wards—were disinclined to speak up, knowing that making a complaint could have serious repercussions. The 1980 DHSS report on the surveys into mixed-sex wards claimed that patients were reluctant to object to mixed-sex arrangements due to ‘embarrassment, fear of seeming ungrateful, and nervousness about possible reprisals’.^120^ One senior (male) charge nurse commented in the 1970s that patients did not like to protest about mixed-sex wards because they would be labelled ‘troublemakers’.^121^

Few patients who did want to complain about abuse were able to break single-handedly through the entrenched system that had so successfully muffled criticism and allegations of violence over decades. Soon after the Second World War patient organisations emerged as part of a general shift towards the recognition of human rights.^122^ Written complaints began to increase during the 1970s as formal processes were established in hospitals. The act of complaining, as Alex Mold has argued, was tied to the notion of the patient as a consumer, even though it was still undermined by the power of the medical establishment.^123^ Furthermore, from the 1970s, psychiatric service-user groups such as the Mental Patients Union were formed, and activists began to expose the abuse of people diagnosed with mental disorders.^124^

Given that male and female patients had previously been segregated in order to prevent pregnancies, new methods of birth control such as the contraceptive pill forced into the open different and difficult questions around the sexual behaviour of patients inside residential institutions. Abortions were legalised in Britain in 1967 and, as we have seen, carried out on some patients in the 1960s and 1970s. From the 1980s, another way of preventing pregnancies in institutions, particularly in those for the ‘mentally handicapped’, was to administer Depo-Provera, a progesterone-based contraceptive delivered by injection and effective for around 12 weeks.^125^ When the AIDS crisis took hold from the mid 1980s, staff could no longer turn a blind eye to sexual behaviour—heterosexual or homosexual, consensual or otherwise. Responsible measures had to be taken by psychiatric service providers to help protect patients not only from sexual assault and unwanted pregnancies, but from contracting sexually transmitted diseases.^126^

### From dangerousness to risk

Some of the new generation of staff and patients who began working in psychiatric units and wards from the late 1970s were able both to ‘see’ abuse and to conceptualise it within emerging discourses around the meaning of sexuality. Added to this was another shift which turned the institutional focus away from the ‘dangerous individual’ (the sexual psychopath) and towards systems of risk. Open-door policies were introduced during the 1950s when, in the words of Nikolas Rose, ‘dangerousness was understood as an internal quality of a pathological individual’. In other words, there was greater focus on managing the dangerous individual than on developing risk-reducing systems that would protect patients. While ‘dangerousness’ made the male psychopath visible to the doctor, I suggest that it rendered the long-stay female patient invisible partly because she was old and not seen to be sexually attractive; partly because she was seen to be passive and did not speak out. ‘Dangerousness’ also made the ‘hyper-sexed’ female psychopath visible—‘she’s a nymph, isn’t she?’, to repeat the words of the nurse quoted above. Rose argued that ‘As one traces the debates through the 1970s and 1980s … dangerousness becomes a matter of factors, of situations, of statistical probabilities’.^127^ Here we see how the emphasis shifted away from the patient and towards systemic risk within the ward or unit.

The hospital abuse inquiries of the early 1970s, together with feminism and patient activism, moved abuse and neglect higher up the clinical and political agenda. By the early 1990s, steps were being taken to raise awareness of patient safeguarding among staff, even though few service providers had developed policies on the subject and women were still being subjected to sexual assaults.^128^ Campaigner Katherine Darton and her colleagues, commented: ‘It seems to many women that professional myopia regarding the general issue of women’s safety could be regarded as a general unthinking refusal to take into account women’s interests and needs in the planning and implementation of mental health services’.^129^

Some clinicians were beginning to speak up too. The anonymous whistle-blowing psychiatrist mentioned above wrote to the *BMJ* in 1988 to share the results of a small survey conducted among 12 women, most of whom had been in-patients on an acute mixed-sex ward. Of this number, he or she reported that ‘five had experienced unsolicited sexual advances while in hospital’; one was the woman who had been raped in a lift. Another woman stated that she had been raped twice and had not felt able to report a vaginal discharge for 18 months. Others recounted how former male patients were returning to the hospital in their cars, picking up female patients and taking them back to their flats where they were forced to have sex. The psychiatrist contended that:

Most staff trained in psychiatry will have witnessed patients becoming uncharacteristically libidinous and promiscuous as a symptom of manic illness. The vulnerability of acutely psychotic patients to unsolicited sexual advances is … recognised less often. It is easy and comforting to dismiss these examples as the elaborated fantasies of crazy patients or to claim that the victims were provocative.

He or she insisted that if nothing was done patients would continue to be ‘at risk’.^130^

That this psychiatrist wrote anonymously to the *BMJ* indicates how contentious the mixing of sexes was. In the early 1990s, the Royal College of Psychiatrists opposed the *Stress on Women* campaign.^131^ A Professor Brice Pitt insisted that women had an ‘ameliorating effect on the men’, maintaining in the following year that sexual abuse was ‘extremely rare’—a claim that was strongly countered by numerous accounts of abuse.^132^

How, then, was it possible to ignore the dangers that some women faced in mixed psychiatric spaces, and what were the mechanisms that began to bring about change? One way of understanding these later historical shifts is to draw on the process of ‘normalisation’, which has been described by Stanley Cohen as taking place when an ‘undesirable situation … is unrecognized, ignored or made to seem normal’. It is then turned into a ‘category of deviance, crime, sin, social problem or pathology’ by a consciousness-raising group. This, he explains, shifts the world of personal suffering into public discourse. It can happen at a micro-level when, in this case, doctors began to ‘see’ the dehumanizing effects of long-term institutionalisation on the individual, even though it had always been present, but hiding in plain sight. And it takes place at the macro societal level, which, I suggest, might refer to broader stigmatised notions of mental patients as being not fully human, not fully sensible (physically and mentally), and not fully sexual. For Cohen, this creates another more supportive narrative, which allows the individual—the female patient—to ‘overcome residual denial, self-blame, stigma or passivity’, whilst making it harder for the offender—psychiatry—to advance its discourse of denial because it would no longer be believed.^133^

## Conclusion

Locking people into psychiatric wards was a cruel and dehumanising practice and there is no doubt that the open-door policies of the 1950s were liberating and necessary. They did, however, have consequences, which included the exposure of female patients to unwanted sexual attention and even rape. I argue that some post-war psychiatrists did not give due consideration to the ramifications of their social experiments. Some were desperate to introduce more therapeutic methods at any cost. Others were swept along by their own professional enthusiasm and hubris. Many were ignorant around matters of sexuality, lacking the imagination and ability to ‘see’ what was in front of them: women who had lived for years in segregated institutions and who were psychologically and socially ill-equipped to protect themselves from sexual harassment and abuse. Ironically, in the mid 1960s, it was male nurses who drew attention to the dangers women faced from male psychopaths, even though the most vocal appear to have been more concerned with protecting their own professional ‘turf’. Opposition to mixed wards gathered pace in the 1970s as patient movements and feminism, which introduced new understandings of sexuality and abuse, called for patients to be given, at the very least, the choice of entering a single- or a mixed-sex ward. By the 1980s, despite greater awareness of the risks of mixed-sex living, adjustments to health care budgets rendered it increasingly difficult to return to segregated wards for financial and practical reasons—a situation which persists to this day.

These, broadly, were the events that unfolded over four decades that witnessed enormous social change, including major shifts in attitudes and behaviours around sexuality, as well as a ‘fragmentation’ in mental health services.^134^ Despite calls for a return to same-sex wards since the 1970s, all governments have failed to meet pledges to abolish mixed-sex wards, and many mental health units are still unable to protect vulnerable patients—both men and women—from sexual violence.^135^ Given the cuts and shortage of resources in the NHS, newspaper headlines referring to breaches in standards on mixed-sex wards look set to continue, deterring some unwell women from seeking help in hospitals where it is still not possible to be ‘ill in peace’.^136^

## Footnotes

1 This was one aim in a broader campaign to end discriminatory practices against women and to improve their rights within mental health care. For a useful account of the campaign, see Kate Mahoney, ‘“Finding Our Own Solutions”: The Women’s Movement and Mental Health Activism in Late Twentieth-Century England’ (unpublished PhD thesis, University of Warwick, 2017), 289–316.

2 The *Daily Mail* campaign has been running for over 20 years. See Sophie Borland, ‘Will the Shame of our Mixed Wards ever End?’, *Daily Mail*, 17 March 2017, 1.

3 MIND, ‘*Stress on Women*, Policy Paper on Women and Mental Health’, 1992, Wellcome Library, Mind archives, PP/EDM/A/3/1/6, 11.

4 On deinstitutionalisation, see Despo Kritsotaki, Vicky Long and Matthew Smith, eds, *Deinstitutionalisation and After. Post-War Psychiatry in the Western World* (Basingstoke: Palgrave Macmillan, 2016); Peter Barham, *Closing the Asylum. The Mental Patient in Modern Society* (London: Penguin, 1992). On the anti-psychiatry movement, see Oisín Wall, *The British Anti-Psychiatrists. From Institutional Psychiatry to the Counter-Culture, 1960–1971* (Abingdon: Routledge, 2018); David Abrahamson, ‘R. D. Laing and Long-Stay Patients: Discrepant Accounts of the Refractory Ward and “Rumpus Room” at Gartnavel Royal Hospital’, *History of Psychiatry*, 2007, 18, 203–15. On therapeutic communitites, see Oisín Wall, ‘The Birth and Death of Villa 21’, *History of Psychiatry*, 2013, 24, 326–40; Catherine Fussinger, ‘“Therapeutic Community”, Psychiatry’s Reformers and Antipsychiatrists: Reconsidering Changes in the Field of Psychiatry after World War II’, *History of Psychiatry*, 2011, 22, 146–63.

5 Vicky Long, ‘“Heading Up a Blind Alley”? Scottish Psychiatric Hospitals in the Era of Deinstitutionalization’, *History of Psychiatry*, 2017, 28, 115–28, 116.

6 John Hopton, ‘Prestwich Hospital in the Twentieth Century: A Case Study of Slow and Uneven Progress in the Development of Psychiatric Care’, *History of Psychiatry*, 1999, 10, 349–69.

7 Fulbourn Hospital is in Cambridge and still operates some wards, Claybury (closed 1997) was in north east London, and Severalls (closed 1997) was in Essex.

8 On the scholarship on child abuse in Britain, see Lucy Delap, ‘“Disgusting details which are best forgotten”: Disclosures of Child Sexual Abuse in Twentieth Century Britain’, *Journal of British Studies*, 2018, 57, 79–107; Louise A. Jackson, *Child Sexual Abuse in Victorian England* (London: Routledge, 2000); Carol Smart, ‘Reconsidering the Recent History of Child Sexual Abuse, 1910–1960’, *Journal of Social Policy*, 2000, 29, 55–71.

9 Claire Hilton, *Improving Psychiatric Care for Older People. Barbara Robb’s Campaign, 1965–1975* (Basingstoke: Palgrave Macmillan, 2017), and Hilton, ‘Whistle-Blowing and Duty of Candour in the National Health Service: A “History and Policy” Case Study of the 1960s and 2010s’, *Journal of the Royal Society of Medicine*, 2016, 109, 327–30.

10 Vicky Long, *Destigmatising Mental Illness? Professional Politics and Public Education in Britain, 1870–1970* (Manchester: Manchester University Press, 2014); ‘“Surely a Nice Occupation for a Girl?” Stories of Nursing, Gender, Violence and Mental Illness in British Asylums, 1914–30’ in Anne Borsay and Pamela Dale, eds, *Mental Health Nursing. The Working Lives of Paid Carers in the Nineteenth and Twentieth Centuries* (Manchester: Manchester University Press, 2015), 131–2.

11 Denis V. Martin, *Adventure in Psychiatry. Social Change in a Mental Hospital* (Oxford: Cassirer, 1962), 81.

12 A survey conducted in Glasgow in the early 1990s concluded that both men and women were the victims of sexual violence on psychiatric wards, see Alison Tonks, ‘Women Patients Vulnerable in Mixed Psychiatric Wards’, *BMJ*, 23 May 1992, 304, 1331; further historical work needs to be done on attitudes and behaviours around issues of race, sexuality and gender identity in respect of sexual violence in psychiatric establishments.

13 Delap, ‘“Disgusting details which are best forgotten”’, 81.

14 I use the terms ‘asylum’, ‘mental hospital’ and ‘psychiatric hospital’ interchangeably.

15 Louise Hide, *Gender and Class in English Asylums, 1890–1914* (Basingstoke: Palgrave Macmillan, 2014).

16
*Ibid*., see ch. 3.

17 Kathleen Jones, *Asylums and After, A Revised History of the Mental Health Services: From the Early 18^th^ Century to the 1990s* (London: Athlone Press, 1993), 136–42.

18 Eric H. Pryor, *A Century of Caring. Claybury 1893–1993* (London: Mental Health Care Group, Forest Healthcare Trust, 1993) 106–8.

19
*Annual Report of the Board of Control to the Lord Chancellor for the Year 1954* (London: HMSO, 1955), 3.

20
*Royal Commission on the Law Relating to Mental Illness and Mental Deficiency 1954–1957. Report* (London: HMSO, 1957), 317.

21 On wartime revelations, see David H. Clark, *The Story of a Mental Hospital. Fulbourn 1858–1983* (London: Process Press, 1996), 39.

22 WHO, ‘The Community Mental Hospital: Third Report of the Expert Committee on Mental Health’ (Geneva: WHO, 1953), 19.

23 Alfred H. Stanton and Morris S. Schwartz, *The Mental Hospital* (London: Tavistock Publications, 1954); see also the well-known study from Erving Goffman, *Asylums. Essays on the Social Situation of Mental Patients and Other Inmates* (New York: Doubleday Anchor, 1961), together with W. Caudill, *The Psychiatric Hospital as a Small Society* (Cambridge, MA: Harvard University Press, 1958), and Ivan Belknap, *Human Problems of a State Mental Hospital* (New York: McGraw Hill, 1956).

24 Russell Barton, *Institutional Neurosis* (Bristol: John Wright & Sons, 1959).

25 John Turner *et al*., ‘The History of Mental Health Services in Modern England: Practitioner Memories and the Direction of Future Research’, *Medical History*, 2015, 59, 599–624, 603.

26 Despo Kritsotaki, Vicky Long and Matthew Smith, ‘Introduction: Deinstitutionalisation and the Pathways of Post-War Psychiatry in the Western World’, in Kritsotaki, Long and Smith, eds, *Deinstitutionalisation and After*, 1–36, 26.

27 Liam Clarke, *The Time of the Therapeutic Communities. People, Places, Events* (London: Jessica Kingsley, 2004), 69.

28 Jones, *Asylums and After*, 157.

29 Shulamit Ramon, *Psychiatry in Britain. Meaning and Policy* (Beckenham: Croom Helm, 1985), 154.

30 N.A., ‘Freedom in Mental Hospitals. The End and the Means’, *Lancet*, 6 November 1954, 964–6, 965.

31 R. J. Roberton, ‘Mixing the Sexes at a Mental Hospital’, *Nursing Times*, 6 March 1959, 285–7, 285.

32 N.A., ‘Freedom in Mental Hospitals’, 964–5.

33 J. C. Barker, ‘Integration in Mental Hospitals’, *Lancet*, 3 July 1965, 37. Barker was responding to sensationalist press reports following the COHSE conference in Aberdeen, referred to below.

34 Roberton, ‘Mixing the Sexes at a Mental Hospital’, 285–6.

35 Clarke, *The Time of the Therapeutic Communities*, ch. 4, and ‘The Opening of Doors in British Mental Hospitals in the 1950s’, *History of Psychiatry*, 1993, iv, 527–51, 549.

36 Jones, *Asylums and After*, 161 (apart from a small rise in 1961), and 187.

37
*Ibid*., 187–8.

38 ‘Interview 13, Peter Cheng, Community Psychiatric Nurse, 16 & 28.8.95’, Wellcome Library, Diana Gittins archive, Severalls Hospital, Essex, 1995, GC/244/2/13, 33.

39 In 1973, 1,985 probation orders were issued to offenders who agreed to go to hospital to avoid a prison sentence. In the same year, 956 offender patients were detained under Part V of the 1959 Mental Health Act in local NHS hospitals without restrictions, 191 with restrictions, and 1,776 were classified as ‘others’ and admitted mainly under Section 136 of the Act when they were taken by the police to a hospital ‘as a place of safety’ if they were ‘in immediate need of care or control’ (DHSS, *Report of the Committee on Mentally Abnormal Offenders,* Cmnd 6244 ((London: HMSO, 1975)), 203, 22, 131).

40 N.A., *Health Services Journal*, February 1968, 21, 16–17.

41 D. H. Clark, ‘Functions of the Mental Hospital’, *Lancet*, 17 November 1956, 1006.

42
*Ibid*., 1008.

43 Cited by Clarke in ‘The Opening of Doors’, 537.

44 Martin, *Adventure in Psychiatry*, 82–3 (emphasis added).

45 ‘Interview 19, Diana Gittins archive, 1995–6, GC/244/2/19, 110.

46
*Ibid*., 110.

47 Diana Gittins, *Madness in its Place. Narratives of Severalls Hospital, 1913–1997* (London: Routledge, 1998), 104. My thanks to Diana Gittins for commenting on this quote.

48 Long, ‘“Surely a Nice Occupation for a Girl?”’, 133.

49 N.A., *Health Services Journal*, June 1968, 21, 17; this was the *Report of the Committee on Senior Nursing Staff Structure* (1966), which was chaired by Brian Salmon and proposed a major restructuring of the roles and responsibilities of nurses in the NHS.

50 N. A., *Health Services Journal*, September 1968, 21, 13.

51 ‘“Blatant Sex” of Patients in Mental Homes’, *The Sun*, 24 June 1965. Newspaper cuttings relating to this section can be found at Warwick University: Modern Records Centre, Confederation of Health Service Employees archive, ‘Press Cuttings’, MSS.229/6/C/CO/10/1.

52 N.A., *Health Services Journal*, August 1965, 18, 16–17, 17.

53 ‘Mental Home Sex. Prostitutes and Birth Pills—All on the National Health’, *Scottish Daily Mail*, 24 June 1965.

54 ‘“Blatant Sex”, *The Sun*.

55 ‘Ward “Sex Orgies” Claim Raised’, *Sheffield Telegraph*, 26 June 1965; ‘Sordid Sex Life in Mental Hospital Mixed Wards’, *Aberdeen Press and Journal*, 24 June 1965.

56 ‘Keep Criminals off Wards’, *Daily Record*, 22 June 1965, 9.

57 ‘“Blatant Sex”’, *The Sun.*

58 ‘Exaggeration. The Danger. Mr Ross on “Sex Orgy” Charges, *The Scotsman*, 26 June 1965.

59 ‘Hospital Sex Shock’, *Scottish Daily Express*, 24 June 1965.

60 Barker, ‘Integration in Mental Hospitals’, 37.

61 The quote is from ‘“Blatant Sex”’, *The Sun*.

62 N. A., *Health Services Journal*, April 1969, 22.

63 Gay Search, ‘Are Mixed Wards Bad Medicine?’, *Woman*, 19 August 1978, 47.

64 ‘Nurses’, The National Archives (TNA hereafter), MH150/583, C.

65 N. A., *Health Services Journal*, September 1968, 21, 13.

66 Martin, *Adventure in Psychiatry*, 81.

67 David Sharpe, ‘Male Nurses on Female Psychiatric Wards’, *Nursing Times*, 11 October 1979, 1773–4, 1773.

68 Letter from Peter D. Mellor to Chief Nursing Officer, RCN, n.d., TNA, MH150/906.

69 Section 128 of the 1959 Mental Health Act stipulated that it was unlawful for a male member of staff to have sex with a woman who was being treated for a mental disorder in hospital.

70 N. A., TNA, MH150/583.

71 Gittins has reported that one female nurse was dismissed for ‘sleeping with a female patient’, Gittins, *Madness in its Place*, 155.

72 John Adams, ‘“Challenge and Change in a Cinderella Service”: A History of Fulbourn Hospital, Cambridgeshire, 1953–1995’ (unpublished PhD thesis, The Open University, 2009), 194 (transcript, 27).

73 ‘“Blatant Sex”’, *The Sun*.

74 ‘Interview 19, Diana Gittins archive, GC/244/2/19, 110.

75 Turner *et al.*, ‘The History of Mental Health Services in Modern England’, 607.

76 Martin, *Adventure in Psychiatry*, 82.

77 N. A., ‘Teach Nurses about Pill—Says Doctor’, *Health Services Journal*, October 1969, 22, 23.

78 For the quote from a nurse, see Gerda L. Cohen, *What’s Wrong with Hospitals* (Harmondsworth: Penguin, 1964), 151. For fears surrounding male patients being assaulted, see ‘Hospital Sex Shock’, *Scottish Daily Express*.

79 Andrew Payne and Sally Hardy, ‘Sexual Activity among Psychiatric Inpatients: International Perspectives. England’, *The Journal of Forensic Psychiatry,* 1993, 4, 110–29, 128.

80 Barton, *Institutional Neurosis*, 76.

81 See Barham, *Closing the Asylum.*

82 Kingston Richmond & Esher Community Health Council. Working Party on the Care of the Elderly. Report on Inpatient Care, July 1976, TNA, MH150/583.

83
*Royal Commission*, 1957, 132.

84 Peter Cryle and Lisa Downing, ‘Feminine Sexual Pathologies in Nineteenth- and Early Twentieth-Century Europe’, *Journal of the History of Sexuality*, 2009, 18, 1–7, 3.

85 See Lucy Bland and Laura Doan, eds, *Sexology in Culture. Labelling Bodies and Desires* (Cambridge: Polity Press, 1998).

86 Carol Groneman, ‘Nymphomania. The Historical Construction of Female Sexuality, *Signs*, 1994, 19, 337–67, 359.

87
*Royal Commission*, 1957, 132.

88 Joanna Bourke, *Rape. A History from 1860 to the Present Day* (London: Virago, 2007), 28–33.

89
*Ibid*., 32.

90 Anonymous, ‘Sexual Drive of Patients in Psychiatric Hospitals’, *BMJ*, 20–7 August 1988, 561.

91 Martin, *Adventure in Psychiatry*, 82 (emphasis added).

92 East Anglian Regional Hospital Board, Fulbourn Hospital, Cambridge, Medical Superintendent’s Annual Report for 1956, 2. Wellcome Library, WX28.BE5 C17F96 56.

93 Clark, *The Story of a Mental Hospital*, 212; women doctors were divided into two categories in the 1960s: the ‘plain ones’ who were ambitious and did not want to get married, and those who did want to marry and opted for general practice, see Rosemary Pringle, *Sex and Medicine. Gender, Power and Authority in the Medical Profession* (Cambridge: Cambridge University Press 1998), 30–1).

94 Bourke, *Rape. A History*, 10.

95 J. C. Barker, the psychiatrist at Shelton Hospital, ‘Integration in Mental Hospitals’, 37. Martin, *Adventure in Psychiatry*, 82.

96 Stanley Cohen, *States of Denial. Knowing about Atrocities and Suffering* (Cambridge: Polity, 2001), 52.

97 For the report of a prostitute, see ‘Hospital Sex Shock’, *Scottish Daily Express*. Bourke, *Rape. A History*, 9.

98
*Daily Mail*, 1 July 1979; David Fletcher, ‘Single-Sex Wards “are preferred by patients”’, *Daily Telegraph*, 22 October 1979, n.p.; Margaret Louden, ‘Mixed-Sex Wards’, *BMJ*, 4 July 1981, 64.

99 Search, ‘Are Mixed Wards Bad Medicine?’, 46–7.

100
*Ibid*., 46.

101 Department of Health, ‘Mixed Sex Wards. Discussion Paper for Meeting with AHA Chairmen’, TNA, MH150/906, 1.

102 Sharpe, ‘Male Nurses on Female Psychiatric Wards’, 1773.

103 Letter from Terence W. Fox to Miss Jowett, 30 June 1977, TNA, MH150/583.

104 For objections from Asian and orthodox Jewish families, see Katherine Darton, Janet Gorman and Liz Sayce, *Eve Fights Back. The Successes of MIND’s Stress on Women Campaign* (London: MIND, 1994), 10.

105 These were prompted by media reports and Barbara Robb’s exposé *Sans Everything: A Case to Answer* (London: Nelson, 1967), see Hilton, *Improving Psychiatric Care for Older People*.

106 Turner *et al*., ‘The History of Mental Health Services in Modern England’.

107
*Ibid*., 608.

108 Inge K. Broverman *et al*., ‘Sex-Role Stereotypes and Clinical Judgements of Mental Health’, *Journal of Consulting and Clinical Psychology*, 1970, 34, 1–7.

109 Phyllis Chesler, *Women and Madness, Twenty-fifth Anniversary Edition* (London, New York: Four Walls Eight Windows, 1997), 93.

110 Two of the most influential were Juliet Mitchell, *Psychoanalysis and Feminism* (New York: Pantheon, 1974); Elaine Showalter, *The Female Malady, Women, Madness and English Culture, 1830–1980* (New York: Pantheon, 1985). See Joan Busfield’s criticism of Showalter in ‘The Female Malady? Men, Women and Madness in Nineteenth Century Britain’, *Sociology*, 1994, 28, 259–77.

111 On the framing of incest, see Bourke, *Rape. A History*, 9. For details regarding its prevalence, see Carol Groneman, *Nymphomania: A History* (London: Norton, 2001), 144–5.

112 Joanna Bourke, ‘Sexual Violation and Trauma in Historical Perspective’, ARBOR Ciencia, *Pensamiento y Cultura*, CLXXXVI 743, May–June 2010, 407–16, 408; see also Jackson, *Child Sexual Abuse in Victorian England*, ch. 8.

113 Joan Busfield, *Men, Women and Madness. Understanding Gender and Mental Disorder* (Basingstoke: Palgrave Macmillan, 1996), 224.

114 Darton *et al*., *Eve Fights Back*, 11.

115 Tonks, ‘Women Patients Vulnerable in Mixed Psychiatric Wards’, 1331.

116 Pringle, *Sex and Medicine*, 150–5; The *Stress on Women* campaign played an important role in compelling all psychotherapists to register with the National Register of Psychotherapists in 1993 (Mahoney, ‘“Finding our own Solutions”’, 307).

117 Busfield, *Men, Women and Madness*, 223.

118 Delap, ‘“Disgusting details which are best forgotten”’, 105.

119 Bourke, *Rape. A History*, 15.

120 DHSS, ‘Draft. Discussion paper for meeting with AHA Chairmen’, TNA, MH150/906, 3.

121 Search, ‘Are Mixed Wards Bad Medicine?’, 47.

122 MIND was established as the National Association of Mental Health in 1946; the Patients Association was formed in 1963; for the most recent history of complaining in health care settings, see Jonathan Reinarz and Rebecca Wynter, eds, *Complaints, Controversies and Grievances in Medicine. Historical and Social Science Perspectives* (Abingdon: Routledge, 2015).

123 Alex Mold, ‘Complaining in the Age of Consumption. Patients, Consumers or Citizens?’ in Reinarz and Wynter, eds, *Complaints, Controversies and Grievances*, 179–196.

124 Turner *et al*., ‘The History of Mental Health Services in Modern England’, 612.

125 Monk, ‘Intimacy and Oppression’, fn 3, 64 in Chapman *et al*., eds, *Sexuality and Relationships in the Lives of People with Intellectual Disabilities. Standing in my Shoes* (London: Jessica Kingsley, 2015).

126 Payne and Hardy, ‘Sexual Activity among Psychiatric Inpatients’, 127.

127 Nikolas Rose, ‘Governing Risky Individuals: The Role of Psychiatry in New Regimes of Control’, *Psychiatry, Psychology and Law*, 1998, 5, 177–95, 178.

128 Darton *et al*., *Eve Fights Back*, 30.

129
*Ibid*., 11.

130 Anonymous, ‘Sexual Drive of Patients in Psychiatric Hospitals’, 561.

131 Mahoney, ‘Finding our own Solutions’, 308–9.

132 Darton *et al*., *Eve Fights Back*, 25–6.

133 Cohen, *States of Denial*, 51–2.

134 Turner *et al*., ‘The History of Mental Health Services in Modern England’, 623.

135 On missed government pledges, see Borland, ‘Will the Shame of our Mixed Wards ever End?’, 1. On vulnerable patients experiencing sexual violence, see Martin Williams, ‘Sexual Violence Soars in UK Hospitals’, *Guardian*, 31 December 2014. <https://www.theguardian.com/society/2014/dec/31/sexual-violence-soars-uk-hospitals>, accessed 16 October 2018.

136 MIND, *Stress on Women*, 11.

## Notes

Dr Louise Hide is a Wellcome Trust Research Fellow in Medical Humanities. Her research project is titled ‘Cultures of Harm in Residential Institutions for Long-term Adult Care, Britain 1945–1980s’. Her first monograph, *Gender and Class in English Asylums, 1890–1914* was published in 2014. She has published on psychiatric institutions, the ‘patient experience’ and the cultural history of pain and syphilis.

